# Isothermal and kinetic screening of methyl red and methyl orange dyes adsorption from water by *Delonix regia* biochar-sulfur oxide (DRB-SO)

**DOI:** 10.1038/s41598-024-63510-0

**Published:** 2024-06-12

**Authors:** Ahmed Eleryan, Mohamed Hassaan, Muhammad A. Nazir, Syed S. A. Shah, Safaa Ragab, Ahmed El Nemr

**Affiliations:** 1 Environment Division, National Institute of Oceanography and Fisheries (NIOF), Kayet Bey, Elanfoushy, Alexandria, Egypt; 2 Institute of Chemistry, The Islamia University of Bahawalpur, Bahawalpur, 63100 Islamic Republic of Pakistan; 3 Department of Chemistry, School of Natural Sciences, National University of Sciences and Technology, Islamabad, 44000 Islamic Republic of Pakistan

**Keywords:** *Delonix regia* biochar, Methyl orange, Methyl red, Adsorption kinetics, Adsorption isotherm, Wastewater treatment, Environmental chemistry, Pollution remediation

## Abstract

In this study, *Delonix regia* seed pods (DRSPs) as a locally available material were refluxed in 90% H_2_SO_4_ to yield a novel *D. regia* seed pods biochar-sulfur oxide (DRB-SO). FTIR, BET, BJH, SEM, EDX, XRD, DSC and TGA were applied to investigate the characterizations of the prepared DRB-SO. Various adsorption parameters like pH effect, dye concentration effect, adsorbent dose, reaction time isotherm and kinetic study were carried out to explain the process of adsorption of methyl orange (MO) and methyl red (MR) onto DRB-SO. Langmuir's adsorption model perfectly explained the adsorption process onto the surface of DRB-SO as a monolayer. The maximum adsorption efficiency of DRB-SO was (98%) and (99.6%) for MO and MR respectively which attained after 150 min with an adsorbent dose of 0.75 g/L. The pseudo-second-order kinetic model best explained the process of adsorption of MO and MR dyes by DRB-SO. The highest observed adsorption amount was as high as 144.9 mg/g for MO dye and 285.7 mg/g for MR dye, comparable with other reported materials based on activated carbon materials. All of the outcomes signposted a prodigious perspective of the fabricated biochar composite material in wastewater treatment. Using the regenerating DRB-SO through an acid–base regeneration process, six cycles of adsorption/desorption were examined. Over the course of the cycles, there was a minor decrease in the adsorption and desorption processes. Also, it was revealed what the most plausible mechanism was for DRB-SO to absorb the ions of the MO and MR dyes.

## Introduction

Synthetic dyes used in large quantities in textiles are physically and chemically most stable and biodegradable compounds, which are toxic to humans as well as to the environment. Thanks to the stability and water solubility, synthetic organic dyes accumulate in wastewater^1–3^. Numerous traditional techniques including aerobic, anaerobic, biological, adsorption, flocculation, flotation, precipitation, oxidation–reduction, photo-reduction and electrochemical were in practice for the decontamination of wastewater^4–7^. However, most of these techniques are not so efficient regarding dye removal performance and cost-effectiveness.

Methyl orange (MO) dye is a synthetic anionic mono azo dye, which is frequently and uninterruptedly used in many industries including textiles^8–10^ in laboratory experiments and other commercial products^11^. This dye is noxious to aquatic animals and plant life^12^. Acute exposure to MO dye increases the risk of quadriplegia, jaundice, cyanosis, heart rate, tissue necrosis, shock and vomiting in humans^13,14^. Hence, it is imperative to eliminate this pigment from polluted water. Similarly, methyl red (MR) dye is also used in many industrial products as coloring agent and ultimately reaches natural water bodies with industrial wastewater^15,16^. It causes ocular and skin sensitivity, irritation of the pharynx or digestive tract and is mutagenic, if inhaled or ingested^17,18^. Therefore the removal of these toxic azo dyes is very crucial for sustaining life on Earth.

Various techniques are in practice for the removal of these and other noxious pollutants from water including, photocatalytic decomposition photo-Fenton, combined electrochemical processes, membrane filtration and adsorption^7,19–22^. Adsorption is the best and most comprehensive technology for the purification of industrial wastewater before disposal to the environment^23^. There are several low-cost adsorbent materials reported for the treatment of wastewater like layered double hydroxides (LDH), silica, activated carbon (AC), chitosan, pomegranate peel, and ferrites^22,24–26^.

However, these low-cost adsorbents did not have satisfactory adsorption efficiency for MO and MR dyes. Consequently, more work is needed in this area to explore other types of adsorbents to eliminate these hazardous pollutants from industrial wastewater.

*Delonix regia* (Flame tree, Royal Poinciana or Flamboyant) is a large ornamental plant which is native to Madagascar^27^. This plant has much medical importance, as its leaves, barks, flowers and seeds have been broadly utilized for anti-appetent, anti-fertility, antifungal, antiulcer insecticidal, anti-inflammatory and cytotoxic activities^28–31^. Recently, *D. regia* was discovered as an adsorbent for the removal of contaminants from polluted water. Vargas et al.^32^ reported the acid dyes (acid red and acid yellow) adsorption with activated carbon (AC) obtained from *D. regia*. Saravanan and co-authors^33^ fabricated surface-modified AC from *D. regia* seed for the removal of reactive yellow dye from an aqueous medium. Utsev et al.^34^ reported methylene blue removal from water by using *D. regia* activated carbon. However, the adsorption results of these *D. regia* based AC were not so good. Therefore it is the need of the hour to do some modifications in AC obtained from *D. regia* to enhance its adsorption performance.

The effects of the physiochemical properties of *Delonix regia* Biochar-SO (DRB-SO) made from *D. regia* seed pods (DRSPs) on the elimination of MO and MR dyes from wastewater are not well studied at this time. Therefore, this study examines how well biochar is generated from *D. regia* seed pods by dehydrating the material with 90% sulfuric acid (H_2_SO_4_) at boiling (~ 280 °C). The purpose of this is to investigate how well the prepared DRB-SO absorbs MO and MR dyes as a highly cost-effective agricultural waste product. The study looks at the effectiveness of DRB-SO absorption of MO and MR dyes in a batch technique under various working circumstances (pH, dosage of DRB-SO, starting concentration of MO and MR dyes, and contact time). The DRB-SO was characterized using XRD, FTIR, SEM, EDX, BET, BJH, DTA, DSC and TGA studies. Additionally, when designing the adsorption process, the kinetic and equilibrium isotherm models for the absorption of MO and MR dyes by DRB-SO were specifically taken into account.

## Materials and methods

### Instrument and materials

*Delonix regia* seed pods (DRSPs) were collected from a local area in Alexandrian and utilized as the raw material to create DRB-SO, an adsorbent substance. The supplier of sulfuric acid (H_2_SO_4_, Purity 98%) was obtained from the Sigma Aldrich, USA. The MO and MR dyes (obtained from Aldrich, USA) concentrations were measured using an analytical Jena digital spectrophotometer (SPEKOL1300 UV/Visible spectrophotometer) in conjunction with 1 cm optical path glass cells, a shaker (JSOS-500) for mixing procedures, and a pH metre (JENCO 6173) for pH surveys. The adsorption–desorption isotherm of DRB-SO was measured in the N_2_ environment. Using an instrument (BELSORP – Mini II, BEL Japan, Inc.), the surface area, pore size and pore distribution of DRB-SO were determined^35,36^. Monolayer volume (*V*_*m*_) (cm^3^ (STP), surface area (S_BET_) (m^2^/g), average pore diameter (MPD) (nm), total pore volume (*p*_*0*_/*p*_*0*_) (cm^3^/g) and energy constant (*C*) values of DRB-S were obtained by modeling of the adsorption–desorption graph. The microporous surface area (*S*_*mi*_), mesoporous surface area (*S*_*mes*_), mesoporous volume (*V*_*mes*_), and microporous volume (*V*_*mi*_) of DRB-S were calculated by the Barrett–Joyner–Halenda (BJH) model. The calculations were carried out with the software of the BELSORP analysis programme. Using the BJH approach, the pore size dispersion was also ascertained from the desorption isotherm^37^. An investigation of the form of the biochar surface was conducted using a scanning electron microscope (SEM; QUALITY 250). Fourier Transform Infrared (FTIR) spectroscopy (VERTEX70) and the ATR unit model V-100 were used to investigate the functional groups on the surface of DRB-S. IR-observable functional groups on the DRB-S surface were identified in the 400–4000 cm^–1^ wavenumber region using FTIR spectroscopy in combination with the platinum ATR unit. Employing the SDT650-Simultaneous Thermal Analyzer apparatus, thermal analyses were conducted at a ramping temperature of 10 °C/min throughout a temperature range of 50–1000 °C.

### DRB-SO preparation

DPSPs were extensively cleansed with tap water many times to remove any dust, and they were thereafter dried in a furnace at 120 °C for twenty-four hours before being ground and pulverized. A total of 140 g of powdered DPSPs was heated at 285 °C in 750 mL of 90% H_2_SO_4_ solution for 10 h, then diluted with distilled water, filtered and then washed with distilled water until pH 7. The DRB-SO was then cleaned with EtOH and dried at 150 °C in a furnace. Biochar with the designation DRB-SO was produced as a consequence of this reaction.

### Batch adsorption experiment

**Figure 1:**
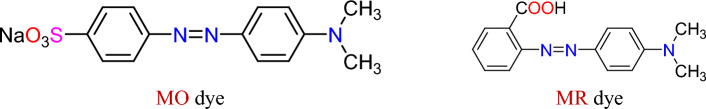
Structure of MO and MR dyes.

A batch adsorption experiment was used to assess the sorption capacity, thermodynamic, and kinetic properties of DRB-SO. A reasonable quantity of flasks with 100 mL of MO (methyl orange, C_14_H_14_N_3_NaO_3_S) and MR (methyl red, C_15_H_15_N_3_O_2_) dyes (Fig. 1) solutions at various starting concentrations and DRB-SO at various weights were shaken for a predetermined amount of time at 200 rpm. Solution pHs were raised or lowered to the appropriate levels with 0.1 M NaOH or HCl. Furthermore, during the adsorption equilibrium investigations, the pH of the solution was maintained at the intended level. Taking a sample (0.1 mL) from the solution at regular intervals (removed from the adsorbent) allowed for the determination of the MO and MR dye concentration using a spectrophotometer set at *λ*_max_ = 505 and 525 nm, respectively. The *q*_t_ of DRB-SO was calculated using Eq. (1).

1 $${q}_{t}=\frac{\left({C}_{0} -{ C}_{t}\right)}{W}V$$

where *C*_*0*_ (mg/L) is the MO and MR dyes initial concentration; *C*_*t*_ (mg/L) is the remaining MO and MR dyes concentration at the end of time *t*; *q*_*t*_ (mg/g) is the adsorption capacity of DRB-SO at time *t*; *W* (g) is the mass of the DRB-SO and *V* (L) is the volume of the MO and MR dyes solutions.

To examine the impact of pH on the adsorption of MO and MR ions by DRB-SO, studies were achieved at different pH values (1.01 to 13.23) and (0.98 to 13.36), respectively, by adding 0.1 g DRB-SO to 100 mL of solutions containing 100 ppm of MO dye and MR. The mixtures were agitated for 150 min. at 200 rpm when the mixtures were at room temperature.

MO dye and MR dye solutions with varying initial concentrations (50–150 ppm) were made, and isotherm measurements and the effect of DRB-SO dose on the adsorption of MO dye and MR dye ions were investigated. Intervals between 0.75 and 1.75 g/L of DRB-SO doses and MO dye and MR dye solutions with diverse starting concentrations were used to measure the MO dye and MR dye concentrations. The mixtures were agitated at 200 rpm and 25 °C. Every adsorption investigation was carried out in triplicate, and the results are presented as an average.

#### Author statement for the use of plants

In this study, Experimental research and field studies on plant material (Orange peels), including the collection of plant waste material, complies with relevant institutional, national, and international guidelines and legislation.

## Results and discussion

### DRB-SO characterization

**Figure 2:**
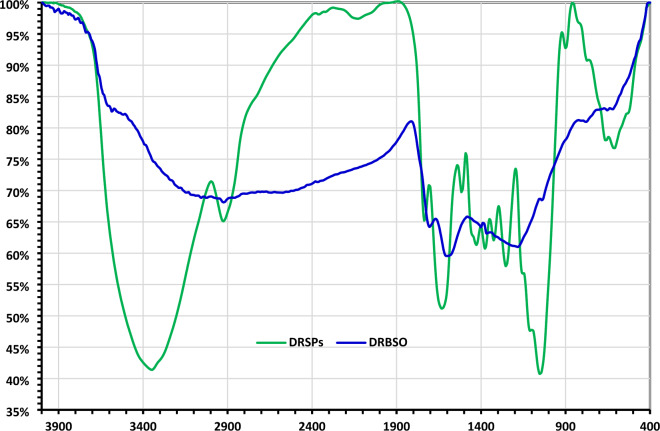
FTIR graphs of (**a**) DPSPs and (**b**) DRB-SO.

Using FT-IR spectroscopy, the functional groups present on the surface of the resulting DRB-SO adsorbent were identified. The FTIR graph of the raw DPSPs and the FTIR graph of the DRB-SO were compared, as shown in Fig. 2. The FT-IR spectra of the materials show changes in their functional groups. The stretching oscillation of the O–H present in the DPSPs and DRB-SO is shown by the band between 3582.15 and 3347.90 cm^–1^ (Fig. 2). The presence of –CH_2_ stretching groups in DPSPs is suggested by the high absorption peaks between 2922.10 cm^–1^ (Fig. 2). These groups were enlarged in DRB-SO and appeared at 2922.56 cm^–1^ (Fig. 2). The C=O stretching of the ester groups in the DPSPs is responsible for the high absorption band at 1733.88 cm^–1^ (Fig. 2). This band was later transformed into a carboxyl group in DRB-SO at 1703.48 cm^–1^ (Fig. 2). Nevertheless, the strength at 1703.48 cm^–1^ increased when DRB-SO was compared to raw DPSPs, indicating that sulphoric acid treatment may increase the carbonyl (C=O) group. The bands at 1627.27 cm^–1^ suggest that the *β*-ketone's C=O stretching oscillation was nearly existent in the DPSPs. This oscillation shifted to 1599.89 cm^–1^ in DRB-SO with high intensity, and it might also be a stretching vibration of –C=C– in DRB-SO (Fig. 2). The DPSPs' C-O functional group is shown by the peaks at 1513.00–1251.19 cm^–1^. This group was replaced by the band at 1405.58 and 1358.18 cm^–1^ in DRB–SO, which displayed the sulfonyl group (SO) stretching vibration (Fig. 2). Additionally, the development of peaks at 1185.98 and 1041.63 cm^–1^ was facilitated by the dehydration process with H_2_SO_4_. These peaks resulted from the production of -SO_3_H and SO groups in DRB-SO. These bands show that the DPSPs treatment with H_2_SO_4_ results in the creation of the DRB-SO. The DPSPs showed a more noticeable rise in the -C–O–C- asymmetric stretching functional group at 1159.21, 1109.16 and 1052.29 cm^–1^ (Fig. 2), compared to DRB-SO, which showed a very weak band at 1041.78 cm^–1^ and two other weak bands at 774.82 and 643.60 cm^–1^^38–41^.

**Figure 3:**
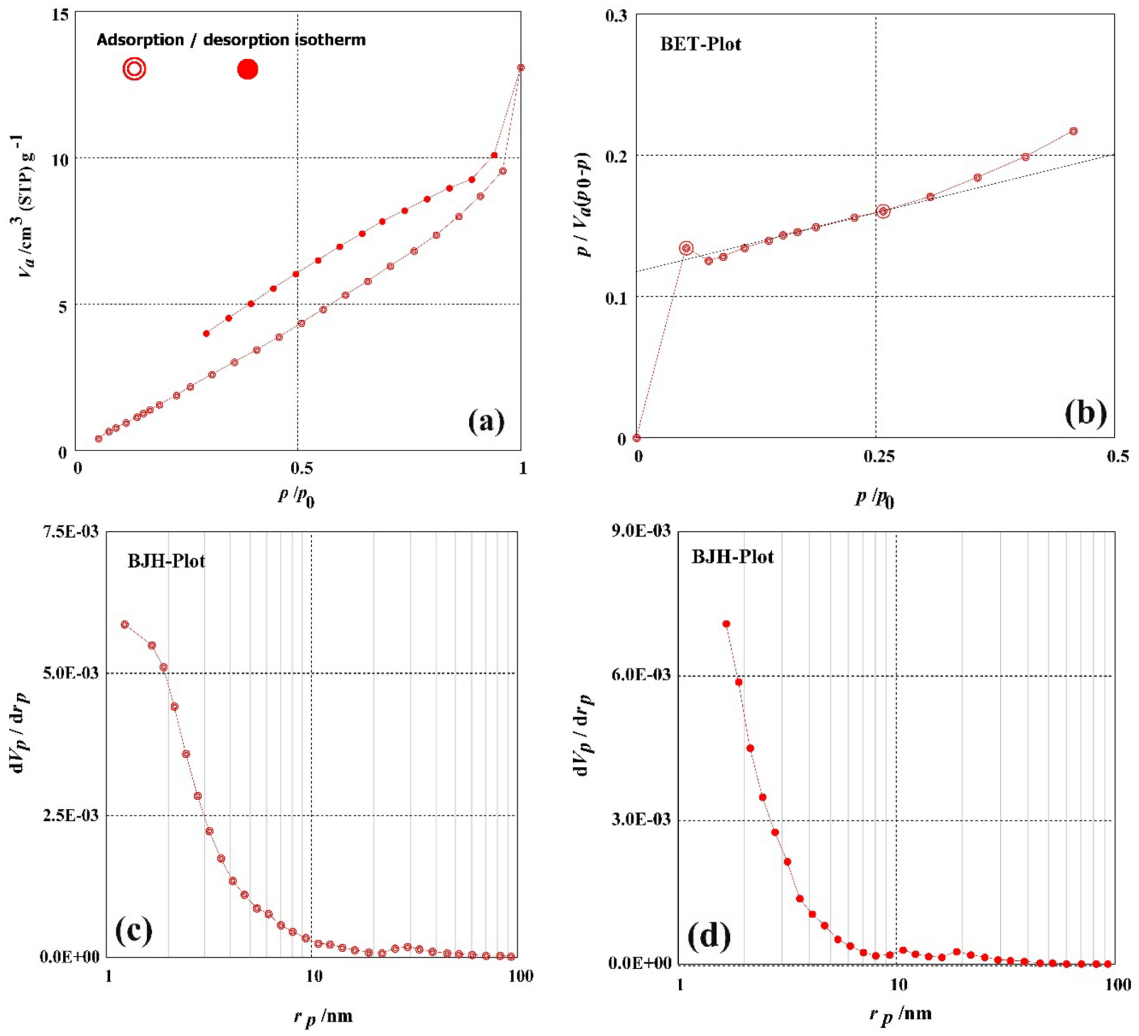
(a) Graph of N_2_ adsorption–desorption, (**b**) graph of the BET, (**c**) graph of the BJH adsorption, (**d**) graph of the BJH desorption of the DRB-SO.

To find out how H_2_SO_4_ affected the DRB-SO's surface characteristics, the N_2_ adsorption–desorption isotherm of the DRB-SO was studied. The specific surface area and mesopore area were calculated using the BET and BJH techniques, respectively. Figure 3 shows the textural properties of DRB-SO, including BET-specific surface area, mass of mesopores, mesopore area, total volume of pores, mesopore distribution peak, average pore diameter, and monolayer volume. The DRB-SO has a relatively tiny BET-specific surface area of 15.39 m^2^/g. DRB-SO had a monolayer volume value of 3.5243 cm^3^ (STP) g^–1^. DRB-SO has a total volume value of 0.1534 cm^3^/g. DRB-SO had mean pore diameters of 4.9481 nm and a total pore volume of 1.8975 × 10^–2^ cm^–1^. The values of 14.823 m^2^/g, 2.1873 × 10^–2^ cm^3^/g, and 1.22 nm were found to be the mesopore volume, meso surface area, and mesopore distribution peak values of DRB-S analysis of adsorption, respectively. The values of 8.3569 m^2^/g, 1.5902 × 10^–2^ cm^3^/g, and 1.66 nm were found to be the mesopore volume, meso surface area, and mesopore distribution peak values of DRB-S analysis of adsorption, respectively.

**Figure 4:**
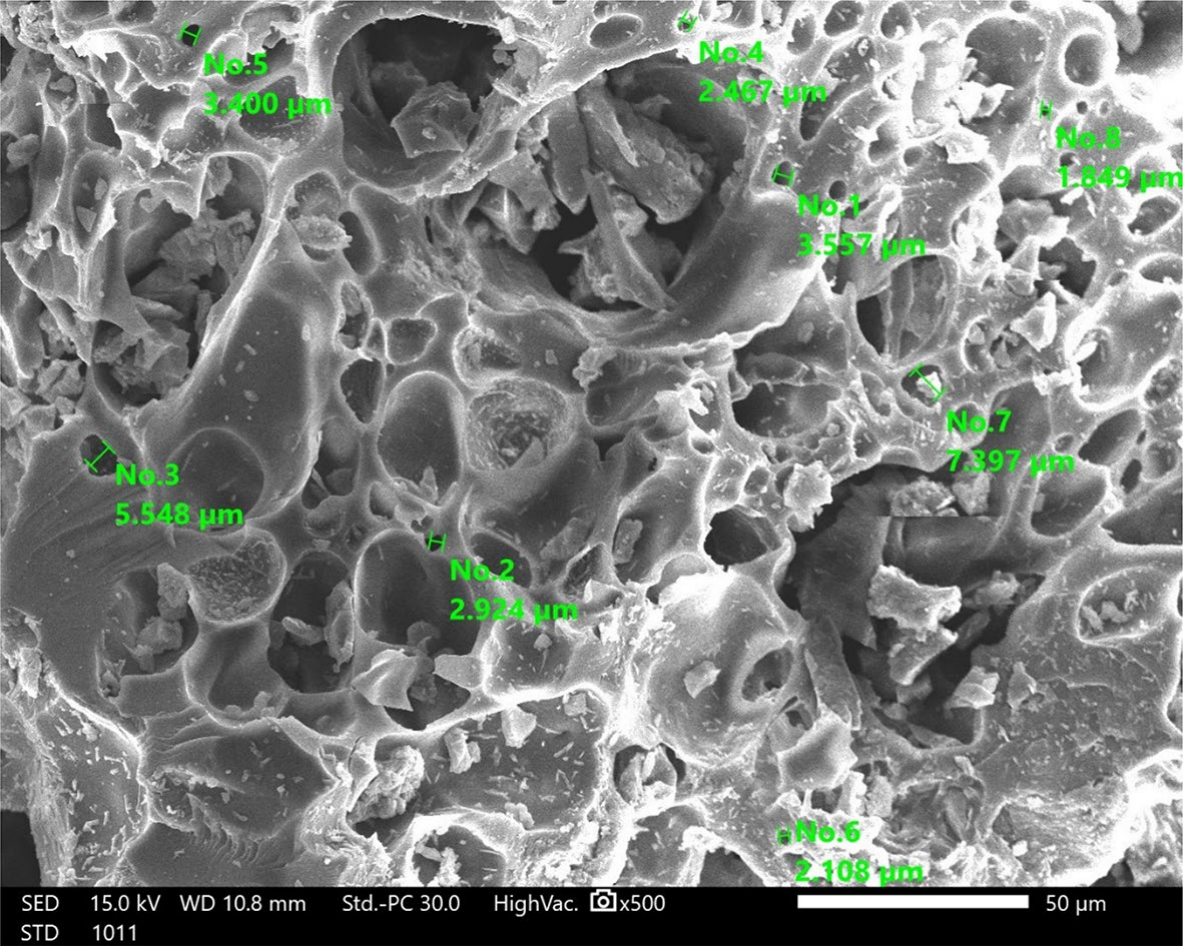
SEM image of DRB-S using High vacuum SEM at magnification × 500 and 15.0 kV.

The DRB-S is shown in SEM pictures in Fig. 4, where it is clear that it is clean and free of impurities. The DPSPs' pore structure remained unharmed by the intense sulfuric acid treatment. The particle size distribution shows that the particle sizes were within the range of 2108–7397 nm and the determined average particle size distribution of the DPKB-SO was 5811 ± 1.65 nm.

The DRB-SO adsorbent chemical composition was studied using scattered X-ray spectrometry (EDX). The percent of each element is presented in Table 1, which indicates that, in addition to carbon mass %, which makes up 58.02% of the sample, there are around 39.63 and 0.66% of oxygen and sulfur, respectively.

**Table 1:** EDX results of prepared DRB-SO.

Elements	DRB-SO	
	Mass%	Atom%
C	58.02 ± 0.50	65.33 ± 0.56
O	39.63 ± 0.98	33.50 ± 0.83
S	0.66 ± 0.07	0.28 ± 0.03
Na	1.28 ± 0.14	0.75 ± 0.08
Ca	0.40 ± 0.07	0.14 ± 0.02
Total	100.00	100.00

**Figure 5:**
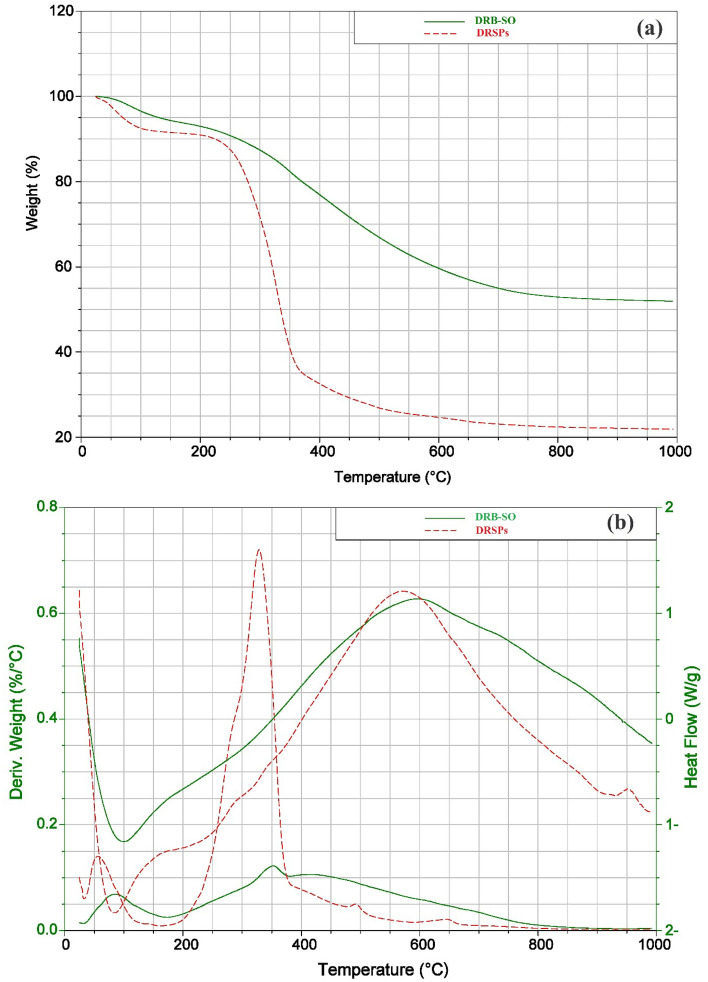
(**a**) Graphs of TGA, and (**b**) graph of DSC and DTA of the DPSPs and DRB-SO.

The impact of structural variations on the operating temperature and degradation behavior of the DRB-SO samples and raw DRSPs were assessed using thermal gravimetric analysis (TGA). Every sample was cooked from 50 to 1000 °C in a N_2_ atmosphere. Figure 5 displays the TGA, Differential Scanning Calorimetry (DSC) and Differential Thermal Analysis (DTA) analytical curves for DPSPs and DRB-SO adsorbent. The first weight reduction was caused by the evaporation of water in the raw DPSPs and DRB-SO, and it peaked before 140 °C. Raw DPSPs and DRB-SO lost weight as a result of the breakdown of many acidic oxygen functional groups that occurred as the temperature rose beyond 140 °C. Moreover, acidic groups break down at different temperatures. For example, phenol breaks down at a greater temperature than lactones, anhydrides, and carboxylic groups. Raw DPSPs exhibit a high weight loss at temperatures up to 330 °C and the final weight loss occurred between 330 and 460 °C. DRB-SO shows three weight losses at temperatures between 25–140, 140–390 and 390–950 °C, which explains the higher stability of DRB-SO compared to the raw DPSPs. TGA curve of DRB-SO converged at temperatures > 410 °C due to carbon breakdown in biomass. At the finishing temperature, various weight loss percentages of 77.03 and 49.06% were obtained for raw DPSPs and DRB-SO, respectively, indicating the greater stability of DRB-SO (Fig. 5a).

DTA graph of DRB-SO and raw DPSPs is illustrated in Fig. 5b. The DTA curve of the raw DPSPs peaked at two points at temperature (*T*_*f*_, 59.10 and 330.30 °C), while the curve of DRB-SO peaked at three points at temperature (*T*_*f*_, 86.40, 352.06 and 456.06 °C) (Fig. 5b). The DTA curve demonstrating the production of DRB-SO adsorbents from raw DPSPs indicates that the dehydration of raw DPSPs yielded two distinct degradation bands. The degradation bands of raw DPSPs decreased from three to two at higher temperatures after treatment with 90% H_2_SO_4_, demonstrating that the degradation degree was intensely affected by treatment with H_2_SO_4_.

Thermal transitions can be employed by DSC to compare materials. Figure 5b depicts the DSC graph of DRB-SO and raw DPSPs. The crystallisation temperatures (*T*_C_) of DPSPs are 73.66 °C, while DRB-SO displays *T*_C_ values was 82.11 °C. When the temperature rises, DRB-SO melts at 587.95 °C, while DPSPs melts at 565.13 °C. A lower *T*_m_ was shown by DPSPs, whereas the highest *T*_m_ was shown by DRB-SO. The grains became more crystalline due to the higher transitional temperatures, which improved both their structural stability and resistance to gelatin disintegration.

**Figure 6:**
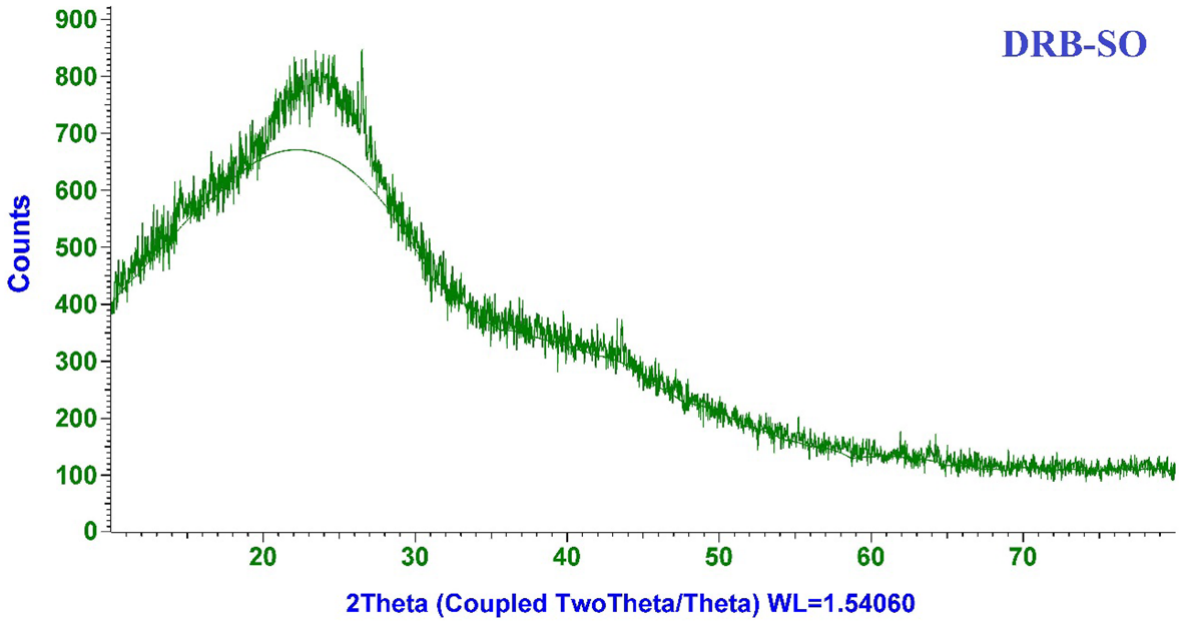
XRD graph of fabricated DRB-SO biochar.

The DRB-SO XRD is shown in Fig. 6 and shows an amorphous carbon structure with arbitrarily oriented aromatic sheets. A tiny peak is located around 2*Ɵ* = 43.54, and a wide peak is indexed as the C (002) diffraction peak in the area of 2*Ɵ* ~ 24.5. A tiny peak is located around 2*Ɵ* ~ 16.4 (101) prominent cellulosic peaks. This might point to a variety of inorganic materials, primarily Quartz and Albite (plagioclase feldspar mineral)^42,43^.

### Effect of pH on dye solution

**Figure 7:**
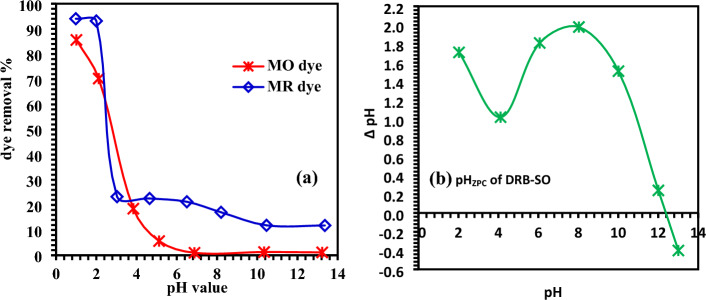
(**a**) pH study for adsorption of methyl orange (100 mg/L) and methyl red (100 mg/L) on *Delonix regia* biochar-sulfur (DRB-SO), (**b**) pH zero point charge (pH_ZPC_) for *D. regia* biochar-sulfur oxide (pH_ZPC_ of DRB-SO).

The pH of the dye solution plays a crucial role in the adsorption process by altering the surface charge of the adsorbent molecule and, eventually, the behaviour of the functional groups that are bound to the adsorbent molecule^44^. The pH effect of MO dye has been examined at various pH values ranging from 1 to 13 with a dye concentration of 100 mg/L (Fig. 7a). The results explored that *Delonix regia* Biochar-Sulfur Oxide (DRB-SO) have maximum adsorption performance in the acidic medium at pH 1.01 (85.6% removal of MO dye) and least performance in the basic medium at pH 13.23 (1.1%). Similarly, the MR dye removal efficiency reached 94% in an acidic medium (pH = 0.98) as compared to a basic medium (11.8% at pH = 13.36). This may be because both MO and MR dyes are basic in nature (Fig. 7a). Because of the strong columbic force that exists between the dye molecules and the positively charged surface of DRB-SO, their adsorptions are highly effective in acidic environments. The adsorption of MO and MR dyes was poor in alkaline media; this might be because the negatively charged surface of DRB-SO and the dye molecules resisted each other^45–47^.

The influence of dye solution pH on adsorbent adsorption efficiency may also be clarified using pH zero point charge (pH_ZPC_). Depending on the functional groups that are connected to the molecule, an amphoteric adsorbent molecule can have both positive and negative charges. The pH of the environment these molecules are in has an impact on their net surface charge. They can become more positively or negatively charged by either receiving or losing protons (H^+^). In the absence of particular sorption, the pH value at which the net charge on a solid surface becomes zero is referred to as the zero point charge^48–50^. In Fig. 7b, the plot of pH versus ΔpH is shown. From the figure pH_ZPC_ of DRB-SO was found to be 12.4. The surface of the adsorbent becomes more positively charged by losing protons when the solution pH is less than pH_ZPC_ (12.5), which promotes the absorption of anionic dyes due to an enhanced electrostatic force of attraction. Adsorption of MO and MR dyes onto DRB-SO was therefore preferred at solution pH values below 12.5 (pH_ZPC_). The equilibrium uptake was low because the adsorbent surfaces resist anionic dye molecules at higher pH values because they become more negatively charged (Fig. 7b).

### Effect of contact time

**Figure 8:**
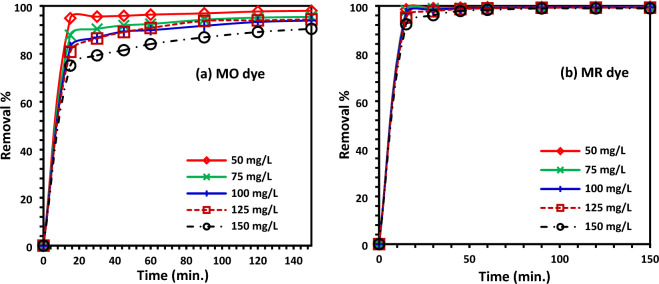
(**a**) % removal of MO dye on DRB-SO at different time intervals using various initial concentrations of MO dye and DRB-SO dose (1.50 g/L); (**b**) % removal of MR dye on DRB-SO at different time intervals using various initial concentrations of MR dye and DRB-SO dose (1.5 g/L).

The adsorption of MO and MR dyes onto *Delonix regia* biochar-sulfur (DRB-SO) was investigated for different reaction times (Fig. 8a,b). For the first half hour, the adsorption rate was greater; as contact time grew, it then decreased and crushed off. The rate of adsorption was high in the beginning because there were more active adsorption sites and space available to transport dye molecules, and the active adsorption sites were empty at that time^51–53^. However, as time goes on, the adsorbate molecules occupy fewer adsorption active sites, which causes the rate of adsorption to gradually decline. From Fig. 8a, it is clear that the maximum adsorption efficiency of *Delonix regia* biochar-sulfur (DRB-SO) was achieved at 150 min (98%) with an adsorbent dose of 0.75 g/L for MO dye. Similarly, for MR dye the maximum adsorption efficiency of *Delonix regia* biochar-sulfur (DRB-SO) was achieved at 150 min (99.6%) with DRB-SO dose 0.75 g/L (Fig. 8b).

### Adsorption kinetics

**Figure 9:**
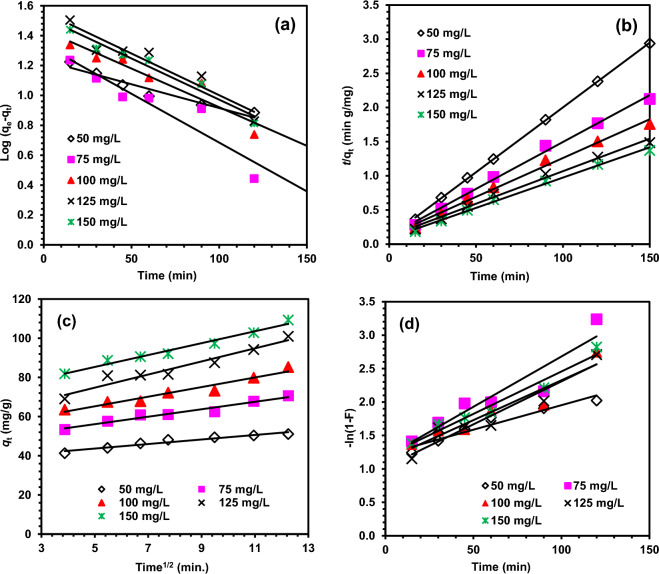
Fitting results of adsorption of MO dye on DRB-SO with adsorbent dose 0.75 g/L, (**a**) pseudo-first-order, (**b**) pseudo-second-order, (**c**) Intrapaticle diffusion model, (**d**) Film diffusion model.

**Figure 10:**
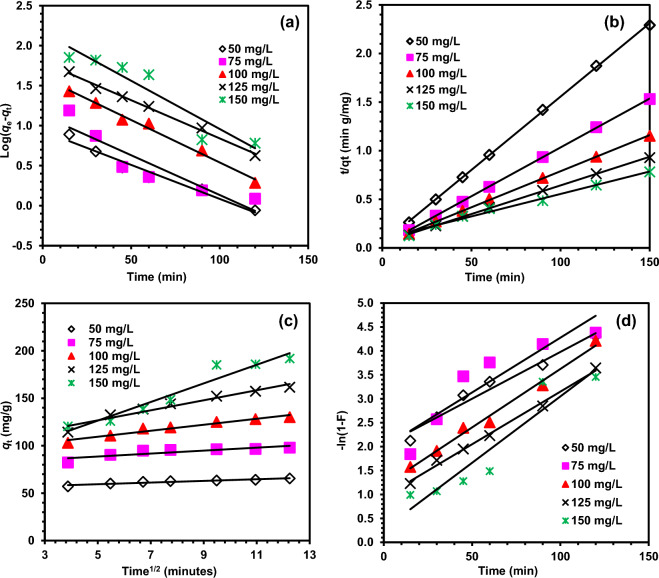
Fitting results of adsorption of MR dye on DRB-SO with adsorbent dose 0.75 g/L, (**a**) pseudo-first-order, (**b**) pseudo-second-order, (**c**) Intrapaticle diffusion model, (**d**) Film diffusion model.

The kinetic study of the adsorption of MO and MR dyes with DRB-SO was carried out by applying pseudo-first-order, pseudo-second-order, intrapaticle diffusion and film diffusion kinetic models on the experimental data (Figs. 9, 10). Table S1–S4 (Supporting data) displayed the kinetic parameters that were derived from these models. For the adsorption of MO dye with DRB-SO, the linear fitting of pseudo first order and pseudo-second-order, intrapaticle diffusion and film diffusion kinetic models, respectively, is shown in Fig. 9a–d^54–57^.

When compared to other kinetic models, the pseudo-second-order has better *R*^2^ values, indicating that it fits the experimental data the best (Fig. 4a–d). Also, the *q*_e_ Cal. (52.63 mg/g) has much closeness to that of *q*_e_ Exp. (64.94 mg/g) with MO dye concentration 50 mg/L and DRB-SO dose 0.75 g/L. similar results were obtained for other concentrations of MO dye and DRB-SO as presented in Table S1. This suggests that the rate of adsorption depends on the adsorption capacity and is not affected by the adsorbate concentration^58–60^. Similarly, the adsorption of MR dye also follows pseudo-second-order kinetic models with relatively higher *R*^2^ value and closed *q*_e_ Cal. (66.23 mg/g) and *q*_e_ Exp. (64.94 mg/g) with MR dye concentration of 50 mg/L and DRB-SO dose 0.75 g/L, and *q*_e_ Cal. (217.39 mg/g) and *q*_e_ Exp. (191.81 mg/g) with MR dye concentration 150 mg/L and DRB-SO dose 0.75 g/L (Table S3).

### Adsorption isotherm study

**Figure 11:**
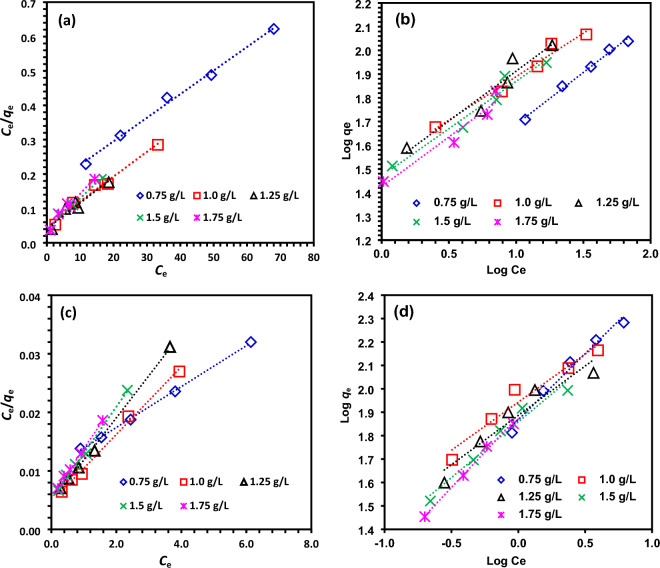
Fitting results for the adsorption of MO dye onto DRB-SO, (**a**) (Linearized Langmuir adsorption Isotherm, (**b**) Freundlich adsorption Isotherm. Fitting results for the adsorption of MR dye onto DRB-SO, (**c**) (Linearized Langmuir adsorption Isotherm, (**d**) Freundlich adsorption Isotherm.

For the real process of adsorbate adsorption onto adsorbent, the adsorption isotherms offer reliable validation (Figure S1)^61–63^. Figure 11 shows the analysis of the adsorption process using the Langmuir and Freundlich isotherm model applied to experimental data. These models' linearized graphs can be used to assess how well these adsorption isotherm models fit. To evaluate each adsorption isotherm model's fitness, the regression coefficient (*R*^2^) is employed as a criteria. Reports were made on the linear form of the Freundlich and Langmuir isotherm model^57,64,65^.

The various parameters related to Langmuir and Freundlich isotherm model are described in Table 2. Rendering to the regression coefficients (*R*^2^ = 0.995 for DRB-SO dose = 0.75 g/L ), the Langmuir isotherm model best pronounced the experimental data, which revealed the adsorption process as a monolayer with uniform adsorption energies may occur in the DRB-SO system for the adsorption of MO dye. Similarly, the adsorption of MR dye onto the surface of DRB-SO also follows the Langmuir isotherm model^66–68^. The maximum adsorption capacity calculated with the Langmuir isotherm model was 144.9 and 285.7 mg/g for MO and MR dyes, respectively (Table 2).

**Table 2:** Fitting results and comparison of the Langmuir and Freundlich Isotherm models constants for different initial MO and MR dyes by varying DRB-SO dose.

Isotherm model	Dye	Isotherm Parameters	DRB-SO dose				
			0.75 g/L	1 g/L	1.25 g/L	1.5 g/L	1.75 g/L
Langmuir	MO	*Q*_*m*_ (mg/g)	144.93	138.89	133.33	108.70	95.24
		*K*_*a*_ × 10^3^	0.0437	0.1463	0.1794	0.2447	0.2770
		*R*^2^	0.9951	0.9770	0.9274	0.9626	0.9612
Freundlich		*Q*_*m*_ (mg/g)	117.8	180.1	235.9	218.6	232.0
		*K*_*a*_	17.7	33.3	31.1	29.5	26.6
		*R*^2^	0.9920	0.9716	0.9323	0.9594	0.9420
Langmuir	MR	*Q*_*m*_ (mg/g)	285.71	172.41	138.89	126.58	121.95
		*K*_*a*_ × 10^3^	0.3	1.2	1.5	1.5	1.5
		*R*^2^	0.9993	0.9949	0.9976	0.9955	0.9976
Freundlich		*Q*_*m*_ (mg/g)	839.7	582.5	589.7	784.7	1764.9
		*K*_*a*_	73.8	87.7	77.0	72.3	76.3
		*R*^2^	0.9787	0.9515	0.9190	0.9646	0.9959

### Comparison of results with reported literature

A selection of earlier research on the removal of MO and MR ions from aquatic medium is shown in Table 3. According to the maximum adsorption capacity (*Q*_m_) reported in this Table, at room temperature, the DRB-SO adsorbent has the highest *Q*_m_ for MR and is equivalent to MO among the literature cited^61,69–77^. For the elimination of MR and MO ions at a given concentration of 1.0 g L^–1^ of DRB-SO adsorbent, these values were 285.71 and 144.93 mg g^–1^, respectively. It is clear from this comparison that the DRB-SO made from DRSPs was a highly effective adsorbent for taking out MR and MO dyes from aqueous solutions.

**Table 3:** The highest amount (*Q*_m_, mg/g) of MO and MR dyes that can be adsorbed onto several documented adsorbent materials.

Adsorbent Material	Dye	*Q*_m_ (mg/g)	References
ZIF-8@SiO_2_@MnFe_2_O_4_	MO	78.12	^69^
MIL-101(Cr)	MO	194	^70^
MgFe-LDO	MO	104.27	^71^
ZIF-67@LDH	MO	180.50	^72^
CuO NPs	MO	121.95	^73^
Ni@ZIF-67	MO	151.74	^61^
DRB-SO	MO	144.93	Current study
Custard apple shell AC	MR	171.23	^74^
Hen feather (HF)	MR	6.02	^75^
CuAl-LDH	MR	209.9	^76^
Ag@Fe nanocomposite	MR	125	^77^
DRB-SO	MR	285.71	Current study

### Regeneration study

**Figure 12:**
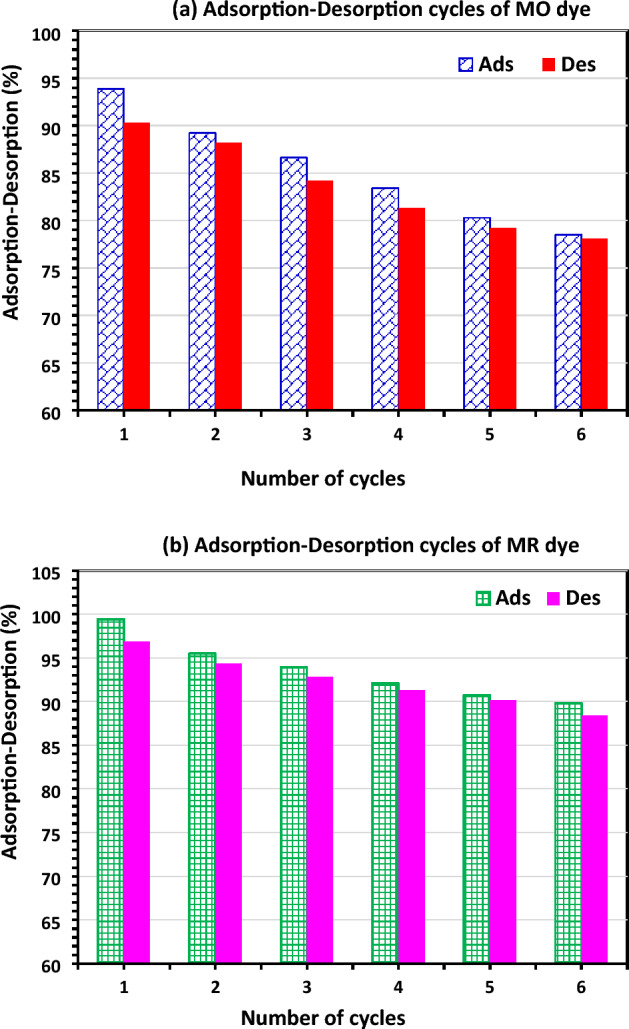
Renewal study of (**a**) MO and (**b**) MR dye adsorption–desorption by DRB-SO adsorbent using dye *C*_0_ (100 ppm) and 1.75 g/L DRB-SO dose at room temperature.

To investigate the feasibility and adsorbent reusability for the absorption of MO and MR dyes, desorption tests of the MO and MR dyes from the DRB-SO adsorbent were done using 0.1 M NaOH as an elution desorption media and its concentrations were measured, and then 0.1 M HCl was used for reactivation of the DRB-SO. In this investigation, when the regeneration cycles increased, the proportion of dye desorption decreased (Fig. 12). Using the regenerated DRB-SO, six cycles of adsorption/desorption have been investigated. Throughout the cycles, the changes in adsorption and desorption were mostly almost constant^78–80^. However, after six cycles, it decreased by about 12.4% for MO dye and 9.6% for MR dye. It suggests DRB-SO could be used as a durable MO and MR dye removal from water (Fig. 12).

### Adsorption mechanism of MB Dye by DRB-S

**Figure 13:**
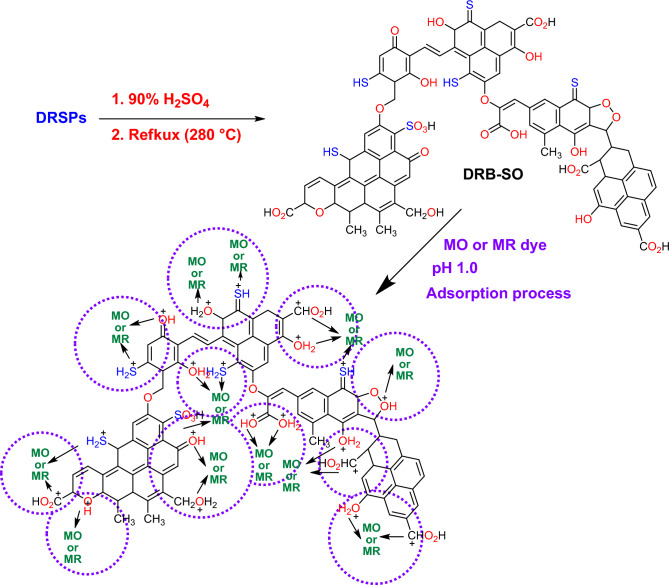
The likely mechanism by which the MO dye or MR dye is adsorbed onto the prepared DRB-SO adsorbent.

Figure 13 explains the likely mechanism via which DRB-SO absorbed the MO dye and MR dye ions. Following the 90% H_2_SO_4_ dehydration of the DPSPs (*D. regia* seed pods raw material). According to FTIR analysis, a variety of functional groups, including C=O, COOH, C–O–C, hydroxyl O–H, C-S, SH, SO_3_H and SO groups, developed on the surface of the adsorbent (DRB-SO). Because of the electrostatic interaction between the sulfur and oxygen lone pair on the DRB-SO surface and the negative charge on the MO dye and MR dye, the adsorption mechanism of the MO dye and MR dye ions in an acidic medium (pH 1.0) can be accomplished through physical interaction. The acidic pH of the acidic medium attracted ions after the surface charge became positive.

In an acidic environment, the surface of DRB-SO picks up a positive charge, which attracts negatively charged dye molecules^81–83^. Furthermore, there is interaction between the functional groups of the positive ions on the DRB-SO's surface and the negative ions in the solution. Furthermore, dye molecules are more soluble at an acidic pH, which makes it easier for them to adhere to the adsorption sites and diffuse through the DRB-SO's pores. Since the acidic pH of DRB-SO is necessary to promote the adsorption of MO and MR dye molecules onto the material, it is a great way to remove colour from industrial effluent. The most important mechanism is the electrostatic interaction-mediated adsorption of ionizable organic molecules to the positively charged surface of the biochar^83^. An aqueous solution's pH and ionic strength determine how well it draws or repels contaminants.^83,84^.

Furthermore, the capacity of organic contaminants in industrial effluent to adsorb is influenced by the pH of the solution^85^. Parshetti et al.'s study^86^ examined the use of food waste-derived biochar in the adsorption of textile colours in wastewater. They found that an alkaline pH enhanced the adsorption of dyes. It was explained by the strong interaction between the positively charged dyes and the negatively charged sites on the biochar surface^86^. It was less successful in adsorbing organic dye, though, because there was an excess of H^+^ at pH 1.5, which competed with the positive charges of the dye^86^. Tsai and Chen^87^ and Xu et al.^88^ have noted that pH has an impact on biochar's capacity to absorb materials. The capacity of organic and inorganic pollutants from industrial effluent to adsorb on biochar is hence influenced by the pH of the solution, which also affects the charged sites.

## Conclusion

Various adsorption parameters like the effect of dye pH, reaction time, dye concentration and adsorbent dose were analysed to evaluate the performance of DRB-SO for the adsorption removal of MO and MR dyes. Results indicate that DRB-SO performed best in the acidic medium as compared to the basic or neutral medium for the removal of MO and MR dyes. Also with 150 min of contact time, the removal percentage was 97.95% for MO dye and 99.60% for MR dye using DRB-SO (initial dye concentration = 50 mg/L, and DRB-S dose = 1.75 g/L). Various kinetic models were applied to the experimental data and the outcomes showed that the pseudo-second-order kinetic model best explains the process of adsorption of dyes by DRB-SO. In other words, over the complete adsorption range, chemical sorption, also known as chemisorption, is the rate-limiting process. Moreover, an analysis of the adsorption isotherm showed that the adsorption data and the Langmuir equation suited each other rather well. For MO and MR dyes, the highest adsorption capacities determined using Langmuir isotherm models were 144.9 and 285.7 mg/g, respectively. According to the study's findings, DRB-SO could be a suitable option for the adsorption of anionic dye found in wastewater.

### Supplementary Information

Supplementary Information.

## Data Availability

The datasets used in this investigation are accessible for review upon request from the paper's corresponding author.
