# Supplementary material for: Isothermal and kinetic screening of methyl red and methyl orange dyes adsorption from water by Delonix regia biochar-sulfur oxide (DRB-SO)
Source: Sci Rep. 2024 Jun 12;14:13585. doi: 10.1038/s41598-024-63510-0 (PMC11169550; doi:10.1038/s41598-024-63510-0)
Supplement: Supplementary file 1 — Supplementary Information. [file 41598_2024_63510_MOESM1_ESM.docx]

Supporting Data

**Isothermal and kinetic screening of Methyl Red and Methyl Orange dyes adsorption from water by *Delonix Regia* Biochar-Sulfur (DRB-SO)**

Ahmed Eleryan^1^, Mohamed Hassaan^1^, Muhammad A. Nazir^2^, Syed S. A. Shah^3^, Safaa Ragab^1^, Ahmed El Nemr^1^*

Table S1. Comparison of the first- and second-order adsorption rate constants and calculated and experimental *q*_e_ values for different initial Methyl Orange dye and DRB-SO concentrations.

| Parameter | | | First-order kinetic model | | | Second-order kinetic model | | | |
| --- | --- | --- | --- | --- | --- | --- | --- | --- | --- |
| DRB-SO Conc. | MO dye  (mg/L) | *q_e_*  (exp.) | *q_e_*  (calc.) | *k_1_* (L/min) | *R^2^* | *q_e_*  (calc.) | *k_2_* × 10^3^ (g/mg min) | *h*  (mg/g min) | *R^2^* |
| 0.75 g/L | 50 | 64.94 | 17.14 | 7.37 | 0.934 | 52.63 | 3.45 | 9560.23 | 1.000 |
|  | 75 | 97.95 | 21.77 | 14.97 | 0.884 | 72.99 | 1.52 | 8103.73 | 0.995 |
|  | 100 | 130.08 | 27.45 | 11.98 | 0.906 | 87.72 | 1.04 | 8006.41 | 0.991 |
|  | 125 | 161.59 | 36.27 | 12.90 | 0.916 | 105.26 | 0.78 | 8628.13 | 0.991 |
|  | 150 | 191.81 | 33.33 | 12.67 | 0.964 | 112.36 | 0.91 | 11454.75 | 0.994 |
| 1.0 g/L | 50 | 49.79 | 6.01 | 25.33 | 0.965 | 48.08 | 9.45 | 21834.06 | 1.000 |
|  | 75 | 74.37 | 15.51 | 23.03 | 0.942 | 68.49 | 2.41 | 11312.22 | 0.998 |
|  | 100 | 99.06 | 17.18 | 17.27 | 0.887 | 87.72 | 2.31 | 17761.99 | 1.000 |
|  | 125 | 122.63 | 21.22 | 9.21 | 0.823 | 108.70 | 2.56 | 30211.48 | 1.000 |
|  | 150 | 146.06 | 34.48 | 11.28 | 0.989 | 121.95 | 13.39 | 199203.2 | 0.999 |
| 1.25 g/L | 50 | 39.78 | 2.75 | 17.27 | 0.966 | 39.06 | 16.47 | 25125.63 | 1.000 |
|  | 75 | 59.58 | 8.56 | 21.19 | 0.945 | 56.50 | 5.66 | 18050.50 | 1.000 |
|  | 100 | 79.33 | 17.84 | 22.80 | 0.920 | 75.19 | 3.11 | 17605.63 | 0.999 |
|  | 125 | 98.84 | 14.71 | 13.36 | 0.841 | 93.46 | 2.52 | 22026.43 | 0.999 |
|  | 150 | 117.08 | 39.76 | 24.64 | 0.976 | 109.89 | 1.16 | 13966.48 | 0.999 |
| 1.5 g/L | 50 | 33.19 | 1.96 | 21.19 | 0.926 | 32.68 | 25.87 | 27624.30 | 1.000 |
|  | 75 | 49.69 | 7.65 | 29.02 | 0.974 | 48.08 | 8.32 | 19230.80 | 1.000 |
|  | 100 | 66.18 | 12.78 | 25.56 | 0.967 | 63.29 | 4.30 | 17211.70 | 1.000 |
|  | 125 | 82.62 | 23.17 | 35.47 | 0.972 | 80.00 | 3.08 | 19685.04 | 1.000 |
|  | 150 | 98.44 | 26.95 | 22.57 | 0.984 | 91.74 | 1.67 | 14044.94 | 0.999 |
| 1.75 g/L | 50 | 28.46 | 0.70 | 20.96 | 0.922 | 28.09 | 35.60 | 28089.90 | 1.000 |
|  | 75 | 42.64 | 0.18 | 28.79 | 0.974 | 41.32 | 11.57 | 19762.80 | 1.000 |
|  | 100 | 56.81 | 9.39 | 25.56 | 0.967 | 54.64 | 5.98 | 17857.10 | 1.000 |
|  | 125 | 70.90 | 17.03 | 35.47 | 0.972 | 68.97 | 4.30 | 20449.90 | 1.000 |
|  | 150 | 84.81 | 19.80 | 22.57 | 0.984 | 80.00 | 2.34 | 14947.68 | 1.000 |

Table S2. Comparison of the interaparticle diffusion and Film diffusion adsorption rate constants and calculated and experimental *q*_e_ values for different initial Methyl Orange dye and DRB-SO concentrations.

| DRB-SO Conc. | MO dye conc.  (mg/L) | Interaparticle diffusion model | | | Film diffusion model | |
| --- | --- | --- | --- | --- | --- | --- |
|  |  | *K_dif_* | *C* | *R^2^* | *K_FD_* | *R^2^* |
| 0.75 g/L | 50 | 0.87 | 55.07 | 0.914 | 0.007 | 0.934 |
|  | 75 | 1.56 | 80.83 | 0.737 | 0.015 | 0.884 |
|  | 100 | 3.16 | 93.68 | 0.952 | 0.012 | 0.906 |
|  | 125 | 5.29 | 100.31 | 0.946 | 0.013 | 0.916 |
|  | 150 | 9.81 | 77.43 | 0.937 | 0.013 | 0.964 |
| 1.0 g/L | 50 | 0.24 | 46.91 | 0.837 | 0.025 | 0.965 |
|  | 75 | 0.57 | 68.24 | 0.759 | 0.023 | 0.942 |
|  | 100 | 1.09 | 87.59 | 0.675 | 0.017 | 0.887 |
|  | 125 | 3.18 | 88.77 | 0.687 | 0.009 | 0.823 |
|  | 150 | 3.84 | 103.79 | 0.817 | 0.011 | 0.989 |
| 1.25 g/L | 50 | 0.13 | 38.45 | 0.750 | 0.017 | 0.966 |
|  | 75 | 0.21 | 57.33 | 0.757 | 0.021 | 0.945 |
|  | 100 | 0.49 | 74.09 | 0.727 | 0.023 | 0.920 |
|  | 125 | 1.06 | 87.59 | 0.757 | 0.013 | 0.841 |
|  | 150 | 2.71 | 87.35 | 0.880 | 0.025 | 0.976 |
| 1.5 g/L | 50 | 0.06 | 32.58 | 0.850 | 0.021 | 0.926 |
|  | 75 | 0.10 | 48.60 | 0.787 | 0.029 | 0.974 |
|  | 100 | 0.25 | 63.54 | 0.726 | 0.026 | 0.967 |
|  | 125 | 0.63 | 75.86 | 0.737 | 0.035 | 0.972 |
|  | 150 | 1.42 | 82.95 | 0.835 | 0.023 | 0.984 |
| 1.75 g/L | 50 | 0.03 | 28.10 | 0.807 | 0.021 | 0.922 |
|  | 75 | 0.06 | 41.95 | 0.732 | 0.029 | 0.974 |
|  | 100 | 0.14 | 55.29 | 0.721 | 0.026 | 0.967 |
|  | 125 | 0.30 | 67.81 | 0.671 | 0.035 | 0.972 |
|  | 150 | 0.59 | 78.69 | 0.704 | 0.023 | 0.984 |

Table S3. Comparison of the first- and second-order adsorption rate constants and calculated and experimental *q*_e_ values for different initial concentration of MR dye and DRB-SO concentrations.

| Parameter | | | First-order kinetic model | | | Second-order kinetic model | | | |
| --- | --- | --- | --- | --- | --- | --- | --- | --- | --- |
| DRB-SO Conc. | MR dye (mg/L) | *q_e_* (exp.) | *q_e_*  (calc.) | *k_1_* (L/min) | *R^2^* | *q_e_*  (calc.) | *k_2_* × 10^3^ (g/mg min) | *h*  (mg/g min) | *R^2^* |
| 0.75 g/L | 50 | 64.94 | 8.59 | 19.58 | 0.967 | 66.23 | 4.83 | 21186.44 | 1.000 |
|  | 75 | 97.95 | 13.41 | 22.80 | 0.844 | 100.00 | 3.48 | 34843.21 | 1.000 |
|  | 100 | 130.08 | 39.95 | 24.41 | 0.989 | 135.14 | 1.18 | 21598.27 | 1.000 |
|  | 125 | 161.59 | 62.53 | 22.11 | 0.993 | 169.49 | 0.65 | 18656.72 | 0.999 |
|  | 150 | 191.81 | 145.85 | 27.87 | 0.892 | 217.39 | 0.23 | 10787.49 | 0.988 |
| 1.0 g/L | 50 | 49.79 | 3.67 | 40.07 | 0.993 | 50.00 | 21.51 | 53763.44 | 1.000 |
|  | 75 | 74.37 | 9.69 | 45.60 | 0.980 | 75.19 | 10.47 | 59171.60 | 1.000 |
|  | 100 | 99.06 | 13.19 | 38.00 | 0.963 | 100.00 | 5.99 | 59880.24 | 1.000 |
|  | 125 | 122.63 | 43.30 | 38.92 | 0.984 | 126.58 | 1.65 | 26455.03 | 1.000 |
|  | 150 | 146.06 | 55.26 | 32.70 | 0.976 | 151.52 | 1.13 | 26041.67 | 1.000 |
| 1.25 g/L | 50 | 39.78 | 1.89 | 40.53 | 0.996 | 39.84 | 51.22 | 81300.81 | 1.000 |
|  | 75 | 59.58 | 4.81 | 49.98 | 0.896 | 59.88 | 28.75 | 103092.8 | 1.000 |
|  | 100 | 79.33 | 5.70 | 33.16 | 0.960 | 80.00 | 12.81 | 81967.21 | 1.000 |
|  | 125 | 98.84 | 12.30 | 30.63 | 0.971 | 100.00 | 5.56 | 55555.56 | 1.000 |
|  | 150 | 117.08 | 46.22 | 37.31 | 0.986 | 120.48 | 1.65 | 23980.82 | 1.000 |
| 1.5 g/L | 50 | 33.19 | 1.05 | 40.30 | 0.896 | 33.22 | 99.56 | 109890.1 | 1.000 |
|  | 75 | 49.69 | 1.08 | 26.95 | 0.948 | 49.75 | 59.41 | 147058.8 | 1.000 |
|  | 100 | 66.18 | 5.94 | 52.05 | 0.876 | 66.67 | 25.57 | 113636.4 | 1.000 |
|  | 125 | 82.62 | 7.75 | 33.62 | 0.943 | 83.33 | 9.66 | 67114.09 | 1.000 |
|  | 150 | 98.44 | 24.28 | 38.92 | 0.950 | 100.00 | 3.61 | 36101.08 | 1.000 |
| 1.75 g/L | 50 | 28.46 | 0.28 | 19.58 | 0.844 | 28.49 | 178.55 | 144927.5 | 1.000 |
|  | 75 | 42.64 | 0.72 | 35.93 | 0.963 | 42.74 | 97.78 | 178571.4 | 1.000 |
|  | 100 | 56.81 | 2.05 | 40.99 | 0.983 | 56.82 | 46.93 | 151515.2 | 1.000 |
|  | 125 | 70.90 | 3.79 | 40.53 | 0.952 | 71.43 | 23.90 | 121951.2 | 1.000 |
|  | 150 | 84.81 | 7.64 | 39.38 | 0.944 | 85.47 | 11.50 | 84033.61 | 1.000 |

Table S4. Comparison of the interaparticle diffusion and Film diffusion adsorption rate constants and calculated and experimental *q*_e_ values for different initial MR dye and DRB-SO concentrations

| DRB-SO dose | MR dye conc (mg/L) | Interaparticle diffusion model | | | Film diffusion model | |
| --- | --- | --- | --- | --- | --- | --- |
|  |  | *K_dif_* | *C* | *R^2^* | *K_FD_* | *R^2^* |
| 0.75 g/L | 50 | 0.873 | 55.073 | 0.914 | 0.020 | 0.967 |
|  | 75 | 1.557 | 80.831 | 0.737 | 0.023 | 0.844 |
|  | 100 | 3.156 | 93.677 | 0.952 | 0.024 | 0.989 |
|  | 125 | 5.285 | 100.310 | 0.946 | 0.022 | 0.993 |
|  | 150 | 9.805 | 77.428 | 0.937 | 0.028 | 0.892 |
| 1.0 g/L | 50 | 0.245 | 46.910 | 0.837 | 0.040 | 0.993 |
|  | 75 | 0.568 | 68.238 | 0.759 | 0.046 | 0.980 |
|  | 100 | 1.091 | 87.591 | 0.675 | 0.038 | 0.963 |
|  | 125 | 3.183 | 88.774 | 0.687 | 0.039 | 0.984 |
|  | 150 | 3.839 | 103.790 | 0.817 | 0.033 | 0.976 |
| 1.25 g/L | 50 | 0.125 | 38.449 | 0.750 | 0.041 | 0.996 |
|  | 75 | 0.207 | 57.332 | 0.757 | 0.050 | 0.896 |
|  | 100 | 0.491 | 74.086 | 0.727 | 0.033 | 0.960 |
|  | 125 | 1.060 | 87.593 | 0.757 | 0.031 | 0.971 |
|  | 150 | 2.707 | 87.354 | 0.880 | 0.037 | 0.986 |
| 1.5 g/L | 50 | 0.055 | 32.580 | 0.850 | 0.040 | 0.896 |
|  | 75 | 0.100 | 48.598 | 0.787 | 0.027 | 0.948 |
|  | 100 | 0.248 | 63.538 | 0.726 | 0.052 | 0.876 |
|  | 125 | 0.630 | 75.860 | 0.737 | 0.034 | 0.943 |
|  | 150 | 1.424 | 82.952 | 0.835 | 0.039 | 0.950 |
| 1.75 g/L | 50 | 0.032 | 28.100 | 0.807 | 0.020 | 0.844 |
|  | 75 | 0.063 | 41.948 | 0.732 | 0.036 | 0.963 |
|  | 100 | 0.145 | 55.287 | 0.721 | 0.041 | 0.983 |
|  | 125 | 0.298 | 67.812 | 0.671 | 0.041 | 0.952 |
|  | 150 | 0.587 | 78.687 | 0.704 | 0.039 | 0.944 |

|  |
| --- |
|  |

**Figure S1**. The relation curve between *q*_e_ and *C*_e_ of (a) MO dye and (b) MR dye at different DRB-SO doses.
